# Development of a Robust High-Throughput Screening Platform for Inhibitors of the Striatal-Enriched Tyrosine Phosphatase (STEP)

**DOI:** 10.3390/ijms22094417

**Published:** 2021-04-23

**Authors:** Lester J Lambert, Stefan Grotegut, Maria Celeridad, Palak Gosalia, Laurent JS De Backer, Andrey A Bobkov, Sumeet Salaniwal, Thomas DY Chung, Fu-Yue Zeng, Ian Pass, Paul J Lombroso, Nicholas DP Cosford, Lutz Tautz

**Affiliations:** 1 Sanford Burnham Prebys Medical Discovery Institute, NCI-Designated Cancer Center, 10901 N Torrey Pines Rd, La Jolla, CA 92037, USA; llambert@sbpdiscovery.org (L.J.L.); mceleridad@sbpdiscovery.org (M.C.); ldebacker@sbpdiscovery.org (L.J.D.B.); ncosford@sbpdiscovery.org (N.D.C.); 2 Sanford Burnham Prebys Medical Discovery Institute, Conrad Prebys Center for Chemical Genomics, 10901 N Torrey Pines Rd, La Jolla, CA 92037, USA; sgrotegut@loxooncology.com (S.G.); pgosalia@sbpdiscovery.org (P.G.); abobkov@sbpdiscovery.org (A.A.B.); sumeet.salaniwal@kinnate.com (S.S.); tchung@sbpdiscovery.org (T.D.C.); fzeng@sbpdiscovery.org (F.-Y.Z.); ian.pass@sbpdiscovery.org (I.P.); 3 Child Study Center, Departments of Psychiatry and Departments of Neurobiology, Yale University, 230 South Frontage Rd, New Haven, CT 06520, USA; paul.lombroso@yale.edu

**Keywords:** protein tyrosine phosphatase, PTPN5, small-molecule screening, neurodegenerative disorders, Alzheimer’s disease

## Abstract

Many human diseases are the result of abnormal expression or activation of protein tyrosine kinases (PTKs) and protein tyrosine phosphatases (PTPs). Not surprisingly, more than 30 tyrosine kinase inhibitors (TKIs) are currently in clinical use and provide unique treatment options for many patients. PTPs on the other hand have long been regarded as “undruggable” and only recently have gained increased attention in drug discovery. Striatal-enriched tyrosine phosphatase (STEP) is a neuron-specific PTP that is overactive in Alzheimer’s disease (AD) and other neurodegenerative and neuropsychiatric disorders, including Parkinson’s disease, schizophrenia, and fragile X syndrome. An emergent model suggests that the increase in STEP activity interferes with synaptic function and contributes to the characteristic cognitive and behavioral deficits present in these diseases. Prior efforts to generate STEP inhibitors with properties that warrant clinical development have largely failed. To identify novel STEP inhibitor scaffolds, we developed a biophysical, label-free high-throughput screening (HTS) platform based on the protein thermal shift (PTS) technology. In contrast to conventional HTS using STEP enzymatic assays, we found the PTS platform highly robust and capable of identifying true hits with confirmed STEP inhibitory activity and selectivity. This new platform promises to greatly advance STEP drug discovery and should be applicable to other PTP targets.

## 1. Introduction

Tyrosine phosphorylation is a key regulatory process in eukaryotic cell physiology [1,2]. Many human diseases are the result of abnormal expression or activation of protein tyrosine kinases (PTKs) and protein tyrosine phosphatases (PTPs) [3,4,5,6]. Targeted therapies that inhibit PTK function have been very successful with >30 tyrosine kinase inhibitors (TKIs) in clinical use [7,8]. Targeting PTPs has been proven difficult, leading to the stigmatization of these enzymes as undruggable. PTPs are the largest class of protein phosphatases with over 100 members in humans [9,10]. They are challenging targets because they have highly conserved active sites, and small molecules that bind the catalytic site often exhibit poor selectivity. In addition, compounds that target the PTP active site typically contain a charged phosphotyrosine (pTyr)–mimicking group that intrinsically prevents PTP inhibitors from crossing cell membranes. As a result of these challenges, the lack of effective PTP probes has impeded progress in the field for years.

Alzheimer’s disease (AD) is a debilitating neurodegenerative disorder characterized by progressive memory loss and steady decline of cognitive functions [11]. The FDA has approved four drugs to treat the cognitive deficits in AD [12]. However, no effective cure or treatment currently exists. The major pathological hallmarks of AD are the aberrant accumulation of the amyloid-β peptide (Aβ) in the form of amyloid plaques and the intracellular formation of hyperphosphorylated tau protein inclusions [13,14]. Aβ peptides are generated from the processing of amyloid precursor protein by β- and γ-secretases. Significant effort has been put into the development of inhibitors of Aβ production [15]. However, targeting γ-secretase has deleterious effects, likely because γ-secretase cleaves other substrates, such as Notch, which are essential for normal biological function [16]. Clearly, alternative approaches are needed [17]. One approach is to target signaling molecules that are involved in the initial synaptic dysfunction, which occurs prior to the loss of neurons. Such a strategy would provide an early treatment option for AD.

**Figure 1:**
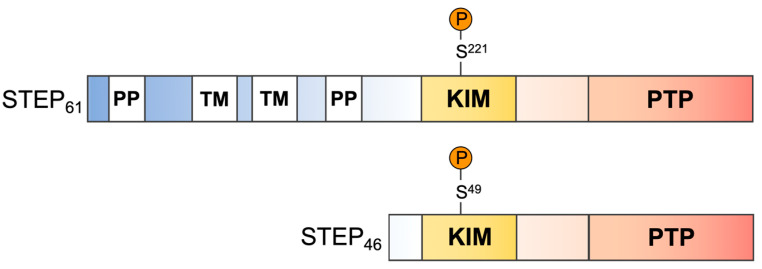
Major STEP splice variants STEP_61_ and STEP_46_. PP, polyproline-rich motif; TM, transmembrane motif; KIM, kinase-interaction motif; and PTP, catalytic domain. Calcineurin dephosphorylation of S221/49 is required for STEP activation.

**Figure 2:**
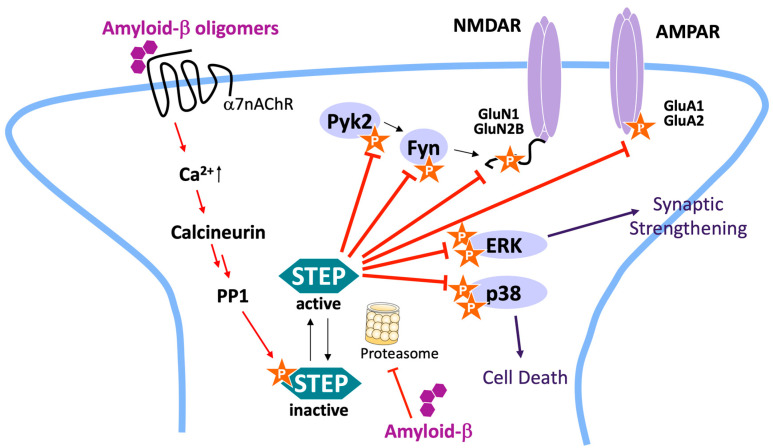
STEP signaling pathways and activation in AD. Amyloid-β (Aβ) oligomers bind to and stimulate α7 nicotinic receptors, leading to Ca^2+^ influx, activation of calcineurin, and dephosphorylation (activation) of STEP by PP1. Aβ oligomers also elevate STEP levels through inhibition of the ubiquitin proteasome system. The net effect is an increase of active STEP. STEP dephosphorylates regulatory Tyr residues in the GluN2B (NR2B) and GluA2 receptor subunits, resulting in internalization of both NMDA and AMPA receptors. STEP also inactivates Fyn, the kinase that phosphorylates GluN2B, directly by dephosphorylation, and indirectly by inactivation of Pyk2, the kinase that activates Fyn. Finally, STEP inactivates MAP kinases ERK and p38, thereby opposing synaptic strengthening and cell death signaling pathways.

Striatal-enriched tyrosine phosphatase (STEP) is a neuron-specific PTP primarily located in postsynaptic terminals of excitatory glutamatergic synapses [18,19]. STEP has two major splice variants, STEP_61_ and STEP_46_ (Figure 1). STEP_61_ is targeted to endo-membranes such as postsynaptic densities (PSDs) by an additional 172 amino acid sequence at its *N*-terminus [20]. This sequence is not found in STEP_46_, which is restricted to the cytosol. Both STEP_61_ and STEP_46_ are equally important for synaptic function but differ in the substrates they preferentially dephosphorylate. Known STEP substrates include the mitogen-activated protein kinase (MAPK) family members extracellular signal-regulated kinases 1/2 (ERK1/2) and p38 [21,22], the tyrosine kinases Fyn and Pyk2 [23,24], the glutamate receptor GluN2B subunit (also known as NR2B) of *N*-methyl-*D*-aspartate (NMDA) receptors [25,26,27], and the GluA2 subunit of α-amino-3-hydroxy-5-methyl-4-isoxazolepropionic acid (AMPA) receptors [27,28,29] (Figure 2). STEP activity is regulated by phosphorylation/dephosphorylation of a Ser residue in the kinase-interaction motif (KIM) [30]. Importantly, the KIM is required for recognition of not only the kinases but for all STEP substrates. STEP levels and activity are increased in the human prefrontal cortex of AD patients, and this is replicated in four mouse AD models [31]. STEP levels and/or activity are also elevated in Parkinson’s disease [32] and in mouse models of schizophrenia [33] and fragile X syndrome [34].

In AD, STEP levels and activity are upregulated by Aβ (Figure 2). Aβ-mediated activation of the calcineurin/protein phosphatase 1 (PP1) pathway leads to activation of STEP [25]. In addition, STEP is ubiquitinated and degraded by the proteasome, and Aβ-mediated inhibition of the proteasome results in increased levels of active STEP [35]. STEP dephosphorylates regulatory Tyr residues on GluN2B and GluA2, and high levels of STEP promote internalization of NMDA and AMPA receptors, respectively [27,36,37]. Lower surface expression of these receptors leads to decreased long-term potentiation (LTP), a form of synaptic plasticity that is closely associated with learning and memory, and increased long-term depression (LTD), a reduction in the efficiency of neuronal synapses. STEP also dephosphorylates Tyr residues within the activation loops of ERK1/2, p38, Fyn, and Pyk2, leading to inactivation of these kinases [21,23,24]. Limited ERK1/2 activation results in decreased transcription of a wide range of signaling molecules important for learning, memory, and protection against neuronal cell death [21]. Inactivation of Fyn and Pyk2 has a more direct effect on NMDA receptor function, as Pyk2 is known to activate Fyn, and Fyn directly phosphorylates the regulatory Y^1472^ on GluN2B that results in its insertion into synaptic membranes. 

The current model of STEP function is that it opposes the development of synaptic strengthening, and that high levels of STEP contribute to the cognitive deficits in AD and other neurodegenerative and neuropsychiatric disorders. Indeed, STEP knockout (KO) mice have enhanced memory and learning abilities [38,39], while genetic reduction of STEP reverses the cognitive and cellular deficits in mouse models of AD, schizophrenia, and fragile X syndrome [27,33,34]. Interestingly, loss of STEP does not alter Aβ or phospho-tau levels [27]. Initial efforts to identify STEP inhibitors resulted in the identification of a STEP tool compound known as TC-2153 [40]. TC-2153 effectively reversed cognitive deficits in AD mice [40], but its benzopentathiepin scaffold reacts with cellular protein thiol groups and modifies DNA [41,42,43,44], precluding this compound from further development. Several high-throughput screening (HTS) campaigns resulted in low micromolar STEP inhibitors (PubChem AID: 588,619 and our unpublished data). However, none of the hits from these screens showed sufficient selectivity for STEP over other phosphatases. Other notable STEP inhibitors include several pTyr substrate-mimicking phosphonic acids with good potency and moderate selectivity for STEP [45]. However, given their charged chemical nature, these compounds are very unlikely to cross the blood–brain barrier. Collectively, prior efforts have not produced a drug-like and selective STEP inhibitor, suggesting that new approaches are needed to identify and advance compounds with properties that warrant clinical development. Here, we report a novel STEP HTS platform that is unique, innovative, and differs from prior STEP HTS strategies by addressing the major weaknesses of previous screening campaigns. This new platform uses a biophysical binding assay based on the protein thermal shift (PTS) technology and utilizes the full-length human STEP_46_ splice variant; the expression and purification of which was optimized to yield the requisite quantities of pure recombinant protein for large-scale screening. This assay platform was found highly robust and capable of identifying true hits with confirmed STEP inhibitory activity and selectivity. 

## 2. Results

### 2.1. Production and Enzymatic Characterization of Recombinant Full-Length STEP_46_

**Figure 3:**
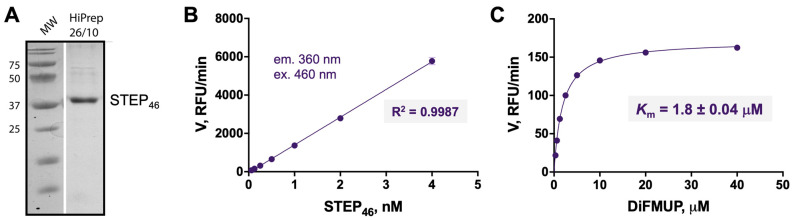
(**A**) SDS PAGE of full-length human STEP_46_ (MW 44.3 kDa) after expression of HIS-tagged fusion protein using a custom-made codon-optimized vector, and purification using Ni-affinity chromatography and subsequent processing over an S75 size exclusion chromatography column. (**B**) Titration of STEP_46_ in a phosphatase activity assay in 384-well format using 6,8-difluoro-4-methylumbelliferyl phosphate (DiFMUP, 50 μM) as a substrate. Fluorescence intensity was measured in kinetic mode to determine the initial rates (*V*) from the slopes of the progress curves. The initial rates were found highly correlative with the various STEP_46_ concentrations tested, as demonstrated by the linear regression coefficient (R^2^). (**C**) Michaelis–Menten kinetics for STEP_46_ (0.5 nM) and DiFMUP using a similar assay format as described in (**B**). The Michaelis–Menten constant (*K*_m_) was calculated by fitting initial rates to the Michaelis–Menten equation using the program GraphPad Prism.

Prior HTS efforts utilized assays with truncated STEP constructs that contained only the catalytic domain. In contrast, we established and optimized a bacterial expression system for the production of full-length human STEP_46_, yielding large quantities of pure protein. The STEP_46_ major splice variant was chosen over STEP_61_ since the latter, containing two transmembrane motifs, was not straightforward enough to express and purify at the scale required for HTS. Codon-optimized human STEP_46_ cDNA was synthesized and cloned into the pET-15b expression vector. HIS-tagged STEP_46_ protein was expressed in *E. coli* and purified via Ni-affinity column chromatography and subsequent S75 size exclusion chromatography to yield ~30 mg of highly pure protein (>95% purity) from a 3 L culture prep (Figure 3A). Next, the enzymatic activity of STEP_46_ was tested in an enzyme titration experiment (Figure 3B). We employed a standard fluorescence intensity phosphatase assay using 6,8-difluoro-4-methylumbelliferyl phosphate (DiFMUP) as the substrate [46,47]. The fluorescence emission readout was highly linear, with STEP_46_ levels tested over a wide range of concentrations. An optimal concentration of 0.5 nM STEP_46_ was determined, yielding initial rates with a signal to background ratio of >50. A kinetic experiment to determine the Michaelis–Menten constant (*K*_m_) for the substrate DiFMUP was performed (Figure 3C). The *K*_m_ for DiFMUP was calculated to be 1.8 ± 0.04 μM (SEM). Taken together, we established a full-length STEP_46_ expression and purification system that produces highly pure, active, and stable protein with excellent yields that allow for the generation of large amounts of recombinant STEP_46_ for HTS purposes.

### 2.2. STEP_46_ Protein Thermal Shift (PTS) Assay Development

**Figure 4:**
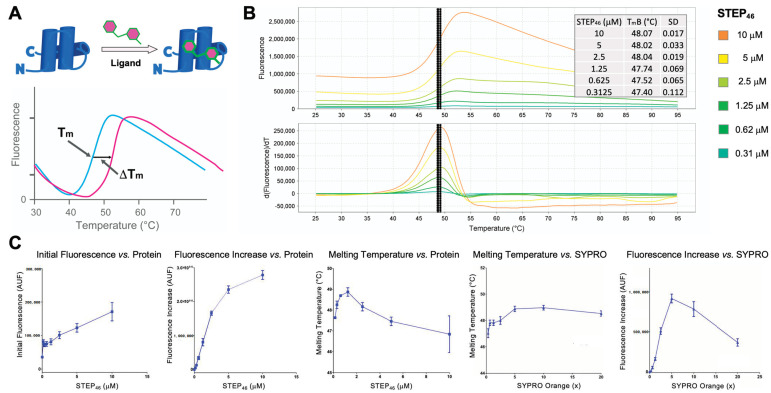
(**A**) Protein thermal shift (PTS) assay principle. Globular protein can be stabilized or destabilized by small-molecule binding, resulting in a change in the protein melting temperature (T_m_). (**B**) STEP_46_ titration in the PTS assay. Melt curves (top) and first derivative plots (bottom) are shown for various concentrations of STEP_46_. Data were recorded using a ViiA 7 thermocycler and analyzed using Protein Thermal Shift software. The STEP_46_ melting profiles demonstrate a single inflection point and a single peak in the first derivative plot, with narrow error margins for the derivative melting temperature. The inset table lists the Boltzmann melting temperature (T_m_B) and standard deviation (SD) for each STEP_46_ concentration tested. (**C**) STEP_46_ PTS assay optimization. The initial fluorescence (F_i_) and fluorescence signal increase (∆FI) for various STEP_46_ concentrations are shown in the two panels on the left. The middle panel shows the measured STEP_46_ T_m_B at various STEP_46_ concentrations. The two panels on the right exhibit the STEP_46_ T_m_B as well as ∆FI at various SYPRO Orange concentrations.

PTS is a biophysical assay that detects the binding of small molecules to a recombinant protein by monitoring its melting temperature (T_m_) (Figure 4A) [48]. To accurately measure STEP_46_ melting temperatures, we employed ThermoFluor technology [48,49], which uses a fluorescent dye, SYPRO^TM^ Orange, that binds to the exposed hydrophobic protein core after melting, resulting in increased fluorescence. A real-time PCR system is used to incrementally heat samples over a temperature gradient and simultaneously measure fluorescence intensity. Small molecules that bind to STEP_46_ are expected to change its melting temperature. Using this technology, we developed a robust STEP_46_ PTS assay in 384-well format (Figure 4B,C). We determined the lowest suitable protein amount per well for HTS applications by comparing the fluorescence signal increase (∆FI) to the initial fluorescence (F_i_). In our experience with PTS assays, a ratio of ∆FI/Fi ≥ 1 ensures a robust HTS assay. We determined amounts of 0.55 μg STEP_46_ per well (1.25 μM in a 10 μL assay volume) to be sufficient. A final dye concentration of 5XSYPRO Orange was found to be optimal. All melting curves demonstrated a single inflection point and a single peak in the first derivative plot, and the T_m_ was relatively invariant of STEP_46_ concentration. Finally, the protein was found to be highly stable on ice and during typical incubation times at room temperature.

### 2.3. STEP_46_ HTS and Hit Confirmation Using the PTS Binding Assay

**Figure 5:**
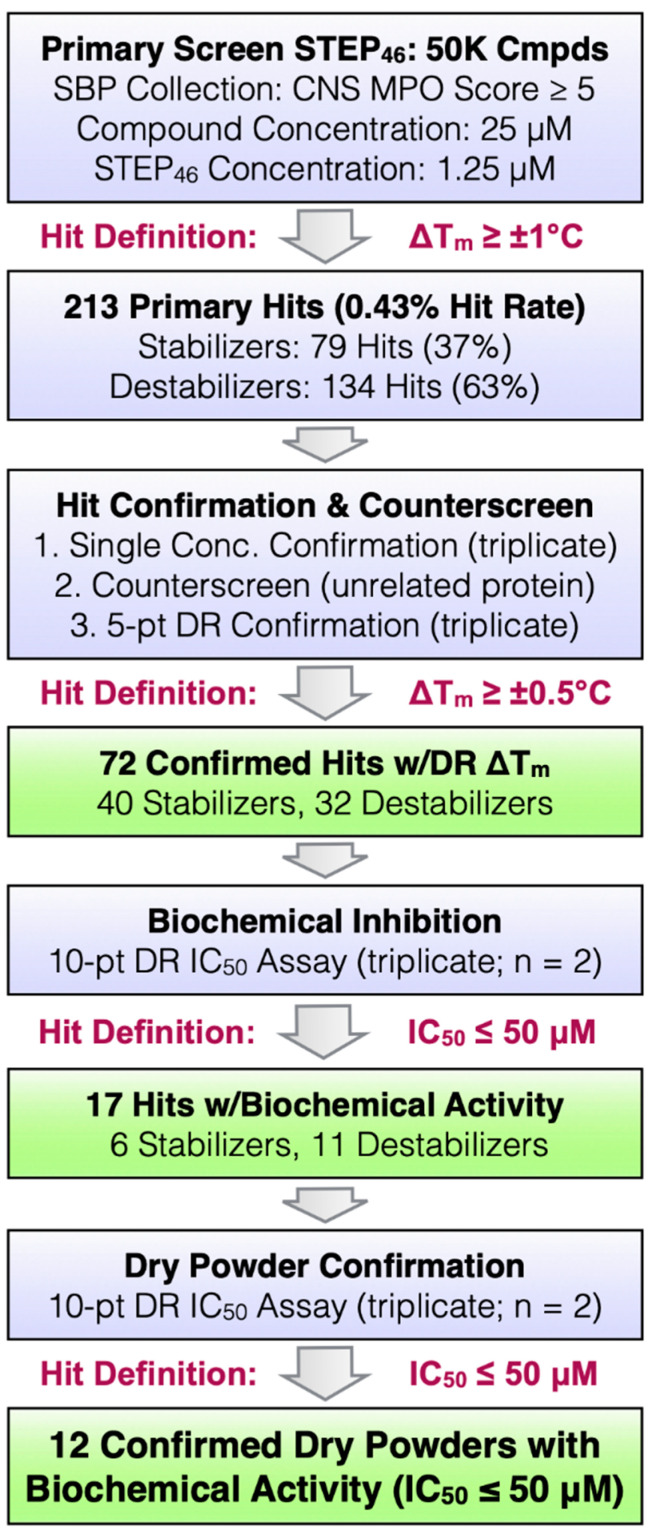
STEP_46_ HTS go/no-go decisions and summary of results.

In order to demonstrate the robustness of the STEP_46_ PTS assay under HTS conditions and to validate the PTS screening approach as a viable alternative to activity-based assays for finding STEP inhibitors, we performed a screen of ~50K small molecules. Go/no-go criteria and a summary of the results at each stage of the testing funnel are shown in Figure 5. Screening compounds were selected from a pool of ~800K small molecules available from our in-house collection. Our selection was based on the central nervous system (CNS) multiparameter optimization (MPO) score [50,51], which predicts the potential of compounds to penetrate the brain. Only compounds with a CNS-MPO score ≥5 were selected for screening. Further, compounds with a lower molecular weight were preferred. An additional cheminformatics filter was used to exclude potential pan-assay interference compounds (PAINS) [52]. 

**Figure 6:**
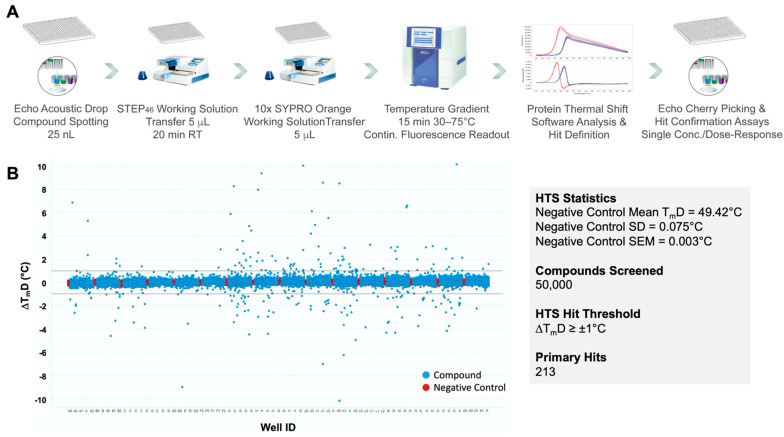
(**A**) Overall HTS workflow and instrumentation. Library compounds are spotted with an Echo 555 Acoustic Liquid Handler into 384-well PCR plates. STEP_46_ and SYPRO Orange working solutions are added using a Multidrop Combi reagent dispenser. A ViiA 7 real-time PCR system is used to incrementally heat samples over a temperature gradient and measure fluorescence intensity. Protein Thermal Shift software is used to analyze the fluorescence raw data and calculate melting temperatures from the first derivative of the melting curve. After hit definition, hit compounds are cherry-picked from compound library plates using Echo, and triplicate single concentration followed by triplicate dose-response PTS hit confirmation assays are performed. (**B**) HTS results and statistics of our 50K small-molecule STEP_46_ PTS primary screen, depicted as ΔT_m_D (melting temperature calculated using the derivative method) distribution vs. plate well ID. The dashed horizontal lines define the hit threshold (ΔT_m_D > ±1 °C).

The overall HTS workflow and instrumentation is shown in Figure 6A. Small-molecule stock solutions or vehicle (DMSO; negative control) were spotted via acoustic dispensing using an Echo^®^ 555 Liquid Handler into 384-well PCR plates. STEP_46_ and SYPRO Orange working solutions were added using a Multidrop^TM^ Combi reagent dispenser. The final library compound concentration was 25 μM. Using a ViiA^TM^ 7 real-time PCR system, fluorescence intensity was followed over 15 min using a temperature gradient of 30 to 75 °C (0.05 °C/s). Fluorescence raw data were analyzed using the Applied Biosystems Protein Thermal Shift^TM^ software. The assay demonstrated excellent statistical values with very narrow error margins (Figure 6B). Using a T_m_ shift of ±1 °C as a hit threshold, 213 primary hits were identified. Primary hits were cherry-picked using an Echo liquid handler and tested in single-concentration (25 μM) confirmation assays in triplicate. To eliminate nonspecific binders, hits were counterscreened against an unrelated protein (lysozyme) using PTS. Finally, STEP-specific binders were tested in a 5-pt dose-response STEP_46_ PTS assay in triplicate. A total of 72 compounds were determined to be specific STEP hits with a dose-dependent T_m_ shift of ≥ ±0.5 °C, corresponding to ≥ 3× standard deviation (SD), or nZ-Score ≥ 3. These compounds were considered as ‘confirmed hits’ and comprised 42 ‘stabilizers’ that increased the T_m_ of STEP_46_ and 32 ‘destabilizers’ that decreased the T_m_ of STEP_46_. Since both stabilizers and destabilizers are known to yield genuine binders/inhibitors [53,54], we progressed all 72 hits into secondary biochemical assays to prioritize compounds for dry-powder acquisition.

### 2.4. Biochemical Characterization of Confirmed STEP_46_ PTS Hits 

**Figure 7:**
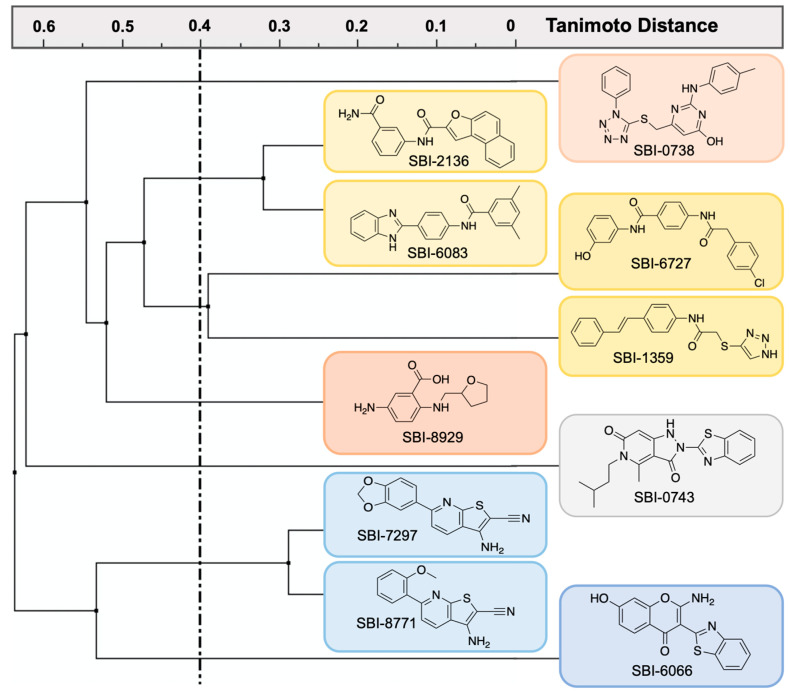
Chemical similarity analysis of 10 powder-confirmed STEP_46_ inhibitors using extended-connectivity FingerPrints (ECFPs). Distance calculations were performed with the ICM Pro software suite (version 3.9, Molsoft, LLC, San Diego, CA, USA).

Employing the established STEP_46_ DiFMUP assay to determine enzymatic activity, we tested the 72 confirmed hits in 10-pt dose-response biochemical inhibition experiments. We found that 17 hits inhibited STEP activity with an IC_50_ value of 50 μM or better. This rate of biochemically active compounds from confirmed PTS hits was in agreement with our prior experience from a similar PTS screening campaign against the SHP2 phosphatase (unpublished data). Next, we obtained commercial dry powders of the 17 biochemically active hits to confirm their activity from pure, fresh material. Powders were quality controlled using standard ^1^H-NMR and LC-MS methods and repurified in cases where purity was determined to be below 95%. Pure powders were then retested in 10-pt dose-response STEP_46_ inhibition assays. Activity could be confirmed for 10 of the 17 powders. Chemical structures of the 10 powder-confirmed hits as well as dose-response curves and IC_50_ values are provided in Table 1. Clustering the 10 hits by chemical similarity analysis using extended-connectivity FingerPrints (ECFPs) [55] revealed seven distinct chemical scaffolds at a Tanimoto distance of 0.4 (Figure 7). Thus, the PTS assay platform yielded diverse chemical matter with a tangible structure–activity relationship (SAR), suggesting that subsequent SAR studies would be fruitful.

One of the major issues in prior STEP HTS campaigns using phosphatase activity as a readout was the lack of potent chemical matter with relative selectivity for STEP (PubChem AID: 588,619 and our unpublished data). Thus, we tested the 10 powder-confirmed hits for their potential to selectively inhibit STEP over the closely related phosphatase PTP1B. For these assays, we expressed and purified the catalytic domain of PTP1B (1-300) using an approach similar to the one described for STEP_46_ above. The enzymatic activity of the highly pure PTP1B was assessed by adapting the DiFMUP phosphatase assay. Similar to STEP_46_, a concentration of 0.5 nM PTP1B was found optimal for kinetic experiments. A Michaelis–Menten kinetic experiment yielded a DiFMUP *K*_m_ value for PTP1B of 25 μM. Using DiFMUP at that concentration, we tested all 10 powder-confirmed hits in 10-pt dose-response inhibition assays against PTP1B. IC_50_ values, as well as dose-response curves, are shown in Table 1. The results demonstrate that several scaffolds selectively inhibit STEP_46_ over PTP1B. Collectively, our HTS data demonstrate the robustness of our STEP_46_ PTS assay and its ability to produce high quality hit compounds with confirmed activity from dry powders.

## 3. Discussion

PTPs, the largest class of phosphatases, are important signaling molecules and potential drug targets in many human diseases. However, targeting PTPs with small molecules has been a challenge because the active site of PTPs is highly conserved and highly charged. Inhibitors that target the active site are often potent but exhibit poor selectivity and bioavailability [46,56,57,58,59]. Indeed, previously reported STEP inhibitors suffer from poor selectivity for STEP and/or lack of efficacy under physiological conditions. Allosteric inhibition of PTPs has recently gained significant traction and has already resulted in potent, selective, and drug-like inhibitors of PTP1B and SHP2 [60]. Our new HTS platform based on PTS technology is designed to identify binders of STEP, including binders of known allosteric pockets [61]. Prior HTS efforts utilized phosphatase activity assays that produced a large number of false-positive hits due to nonspecific STEP inactivation. The main reason for the high false-positive rate is an extremely nucleophilic cysteine (common to all PTPs; p*K*_a_ between 5 and 5.5) [10], which is essential for PTP activity but is easily oxidized or otherwise modified by trace impurities found in library compound collections. In addition, prior HTS campaigns have utilized truncated forms of STEP protein containing only the catalytic domain. Importantly, recent success stories for PTP1B and SHP2 have proven that protein regions outside the PTP domain active site can be effectively targeted with potent and selective allosteric inhibitors [62,63]. 

We chose full-length human STEP_46_ for screening, as STEP_61_, the other major splice isoform, which contains two additional poly-proline and transmembrane motifs (Figure 1), could not be expressed in quantities needed for large-scale HTS. Due to the additional *N*-terminal amino acids in STEP_61_, the two isoforms have distinct substrate specificity in a cellular context (Figure 2). Nonetheless, we do predict that inhibitors of STEP_46_ are likely also to inhibit STEP_61_. The two minor alternatively spliced variants of STEP, STEP_38_, and STEP_20_ were not considered, as they do not contain the consensus PTP domain and therefore are catalytically inactive [20]. We established a biophysical HTS platform for full-length human STEP_46_ based on protein thermal shift technology, allowing the detection of molecules that bind to regions outside the PTP domain such as the KIM. Since PTS requires relatively substantial amounts of pure recombinant protein (~0.5 μg per reaction), we optimized the expression and purification of full-length STEP_46_ to a standard sufficient for large-scale HTS campaigns. Importantly, the robust PTS assay was highly capable of identifying true hits with confirmed activity in STEP biochemical inhibition assays. The primary and confirmatory hit rates were well within the typical range for HTS. Not all binders are necessarily inhibitors of STEP activity or function. However, true binders that do not inhibit the enzyme may have value for alternative drug discovery approaches such as proteolysis targeting chimeras (PROTAC). Critically, the majority of the hits with inhibitory activity passed the dry-powder confirmation stage, resulting in a far greater number of true hits with biochemical activity than previous screens that relied on catalytic activity as the primary readout (PubChem AID: 588,619 and our unpublished data). We found that both stabilizers and destabilizers yielded genuine PTP inhibitors, which is in agreement with other reports in the literature [53,54]. Importantly, several of the newly identified small molecules exhibited relative selectivity in inhibiting STEP_46_ over the closely related phosphatase PTP1B. These scaffolds provide a good starting point for future SAR and optimization studies. 

Finally, the recent failures of γ-secretase inhibitors in clinical trials suggest that other therapeutic approaches in AD are urgently needed. The hypothesis that STEP inhibitors may have value for the treatment of AD shifts the therapeutic objective from reducing Aβ levels to inhibiting a downstream target of Aβ. STEP KO mice are viable, fertile, and appear healthy [38], suggesting that the discovery of specific STEP inhibitors could provide a novel, disease-modifying treatment paradigm not only for AD but also for several other neurodegenerative and psychiatric disorders. Allosteric inhibitors of the tyrosine phosphatase SHP2, a close relative of STEP, are currently in phase I/II clinical trials for the treatment of cancer. The fact that these compounds are highly specific for their target and very drug-like in nature has sparked increased interest in targeting other PTPs in human disease. Interestingly, STEP activity is also regulated through an allosteric mechanism [61], warranting the search for compounds that inhibit STEP via modalities that do not involve the conserved active site. As we have recently shown for SHP2, the protein thermal shift assay is highly competent in detecting the binding of allosteric inhibitors [64]. Thus, our HTS platform should be capable of finding first-in-class allosteric inhibitors of full-length STEP. Importantly, the PTS assay, using ThermoFluor technology, is easily scalable. The critical consideration is the production of pure and well-behaved target protein in amounts needed for HTS (typically between 0.5 and 1 μg/well of purified protein). With our optimized expression and purification procedure for STEP_46_, the screening of significant larger compound collections is conceivable. It is also reasonable to expect that the PTS assay can be applied to other phosphatase targets. In fact, we recently completed a similar screen for SHP2, in which we identified a number of validated scaffolds (Reference [64] and our unpublished data). Therefore, we suggest that our HTS platform using PTS technology has the potential to significantly transform drug discovery efforts not only for STEP but should be widely applicable to other phosphatase targets in the search for novel treatments for many serious human ailments.

## 4. Materials and Methods

### 4.1. Protein Expression and Purification

Codon-optimized human full-length STEP_46_ cDNA was synthesized (GenScript, Piscataway, NJ, USA), cloned into the pET-15b expression vector, and expressed as an *N*-His tagged fusion protein. For expression, transformed BL21 (DE3) cells were cultured and induced with 0.7 mM IPTG for 16 h at 24 °C. Collected cells were resuspended in lysis buffer (25 mM Tris pH 7.5, 300 mM NaCl, 50 mM imidazole, 10% glycerol) with 100 mg/L RNaseA and were lysed with two passages using an EmulsiFlex-C3 microfluidizer (Avestin Inc., Ottawa, Canada). The lysate was clarified by centrifugation at 15,000× *g* for 50 min and applied to HiTrap Ni-NTA resin. The column resin was washed with lysis buffer, and then the STEP protein was eluted in lysis buffer at 300 mM imidazole. The STEP protein was further purified by S75 size exclusion chromatography in 50 mM Tris, pH 7.5, 50 mM NaCl. The eluted peak fractions were supplemented with tris(2-carboxyethyl)phosphine (TCEP) to 10 mM, concentrated by ultrafiltration, and stored at −80 °C. Human PTP1B catalytic domain (1–300) was cloned into PET-15b and expressed as an *N*-His-tagged fusion protein in a manner similar to that described above for STEP_46_.

### 4.2. Protein Thermal Shift Assays (PTS)

Protein thermal shift assays (also known as differential scanning fluorimetry) were adapted and optimized according to methods previously described [64,65]. In brief, compounds were spotted into MicroAmp^TM^ 384-well real-time PCR plates (#4483285, Applied Biosystems, Foster City, CA, USA) using an Echo 555 liquid handler (Beckman Coulter, Indianapolis, IN, USA). STEP working solution (5 μL of 2.5 μM in 50 mM Tris-HCl pH 7.5, 50 mM NaCl, and 5 mM DTT) was added to each well using a Multidrop Combi Reagent Dispenser (Thermo Fisher Scientific, Waltham, MA, USA). In addition, 5X SYPRO Orange (5 μL, Invitrogen/Thermo Fisher Scientific, Carlsbad, CA, USA) dissolved in molecular grade water was equally dispensed into the PCR plate wells, diluting the enzyme solution 1:2. The plate was then sealed with MicroAmp Optical Adhesive Film (Applied Biosystems, Foster City, CA, USA) and spun to collect the reaction mix at the bottom of the plate. Plates were measured using a ViiA 7 Real-Time PCR instrument (Applied Biosystems, Foster City, CA, USA) and a 15 min temperature gradient with a temperature increase of 0.05 °C/s. The melting temperatures according to Boltzmann (T_m_B) or derivative (T_m_D) methods and thermal profiles were determined as described previously using Protein Thermal Shift Software (version 1.3, Applied Biosystems, Foster City, CA, USA) [64,65]. 

### 4.3. Selection of 50K Small Molecules for HTS

An in-house collection of ~800K small molecules was used as the basis for the selection of screening compounds devoid of PAINS [52] and frequent hitters and predicted to have robust alignment of absorption, distribution, metabolism, and excretion (ADME) attributes and suitable brain penetration according to the CNS-MPO desirability score (CNS-MPO ≥ 5) [50,51]. Compound parameters for CNS-MPO calculations were computed using ChemAxon (version 20.11.0, https://www.chemaxon.com) and included the calculated partition coefficient (ClogP), the calculated distribution coefficient at pH 7.4 (ClogD), the topological polar surface area (TPSA), the molecular weight (MW), the number of hydrogen bond donors (HBD), and the acid dissociation constant (pKa). CNS-MPO scores were calculated using the method reported by Wager et al. [51] implemented in Pipeline Pilot (Dassault Systèmes). Potential PAINS were eliminated using a PAINS substructure filter according to PAINS scaffolds reported by Baell et al. [66] and implemented in Pipeline Pilot. Frequent hitters were determined based on previous in-house full-deck (>100,000 compounds) PTS screens of over 15 different targets.

### 4.4. STEP_46_ HTS of 50K Small Molecules

PTS measurement of STEP_46_ protein was performed using optimized methods as described above. The reactions were prepared in a 384-well plate format by combining STEP protein with compounds (final 25 µM) with thermal shift dye and buffer to a final assay volume of 10 μL. Test compounds were spotted in 25 nL (10 mM) into MicroAmp 384-well real-time PCR plates (catalog no. 4483285; Applied Biosystems, Foster City, CA, USA) using an Echo 555 liquid handler. In addition, 5 μL of STEP working solution (3.0 μM STEP in 25 mM Bis-Tris, pH 6.8, 150 mM NaCl, and 5 mM DTT) were added to each well using a Multidrop Combi reagent dispenser (Thermo Fisher Scientific, Waltham, MA, USA). Next, 5 μL of 5X SYPRO Orange (Invitrogen/Thermo Fisher Scientific) dissolved in 25 mM Bis-Tris, pH 6.8, 150 mM NaCl, and 5 mM DTT was equally dispensed into the PCR plates diluting the enzyme solution 1:2. The plates were then sealed with MicroAmp optical adhesive film (Applied Biosystems, Foster City, CA, USA) and spun to collect the reaction mix at the bottom of the plate. The plates were analyzed using a ViiA 7 real-time PCR instrument (Applied Biosystems, Foster City, CA, USA) and an 8 min temperature gradient with a temperature increase of 0.1125 °C/s. T_m_D, and thermal profiles were determined using the Protein Thermal Shift software (version 1.3, Applied Biosystems, Foster City, CA, USA). The threshold for primary hit selection was ΔT_m_D ≥ ±1 °C. Primary hits were cherry-picked from compound library plates using an Echo 555 liquid handler. Hit confirmation assays included triplicate PTS of primary hits at a single concentration (25 μM), followed by a dose-response (100, 50, 25, 12.5, and 6.25 μM). An nZ-Score ≥ 3 was used as a threshold for hit confirmation. Specificity of PTS binding was assessed by testing hits against an unrelated protein (lysozyme) at a single concentration (25 μM).

### 4.5. STEP_46_ and PTP1B Michaelis-Menten Kinetic Assays

Human STEP_46_ and PTP1B activity was measured at room temperature (RT) in a 384-well plate format standard phosphatase fluorescence intensity assay using DiFMUP as a substrate and a total reaction volume of 25 μL. PTP working solutions were prepared at a 0.625 nM concentration (for a final concentration of 0.5 nM) in buffer containing 50 mM Bis-Tris pH 6.0, 50 mM NaCl, 5 mM DTT, and 0.01% Tween^®^ 20. DiFMUP working solutions at 5X final concentration were prepared in 50 mM Bis-Tris pH 6.0, 50 mM NaCl, and 0.01% Tween 20. In addition, 20 μL of PTP working solution was dispensed into a black Greiner FLUOTRAC^TM^ 200 384-well microplate (#781076, Greiner, Frickenhausen, Germany) in triplicate using the Multidrop Combi Reagent Dispenser. The reaction was initiated by addition of 5 μL DiFMUP working solutions for final DiFMUP concentrations of 80, 40, 20, 10, 5, 2.5, 1.25, 0.625, and 0.3125 μM. Fluorescence intensity was measured in kinetic mode (every minute for 10 min) using a Tecan Spark^®^ Multimode Microplate Reader (Tecan, Groedig, Austria) with an excitation wavelength of 360 nm and an emission wavelength of 460 nm. The initial rates were determined from the linear progress curves of the PTP reaction. The nonenzymatic hydrolysis of the substrate was corrected by using a control without addition of enzyme. Michaelis–Menten plots were generated for each PTP construct, and Michaelis–Menten constants (*K*_m_) were calculated using the program GraphPad Prism (version 8, GraphPad Software, Inc., San Diego, CA, USA).

### 4.6. STEP_46_ and PTP1B Biochemical Inhibition Assays

Hit compounds were tested at room temperature (RT) in a 384-well plate format standard phosphatase fluorescence intensity assay using DiFMUP as a substrate and a total reaction volume of 25 μL. Compounds or vehicle (DMSO) were spotted in triplicate into a black Greiner FLUOTRAC 200 384-well microplate for a 10-point dose-response assay using the Echo 555 liquid handler. PTP working solutions were prepared at a 0.625 nM concentration (for a final concentration of 0.5 nM) in buffer containing 50 mM Bis-Tris pH 6.0, 50 mM NaCl, 5 mM DTT, and 0.01% Tween 20. DiFMUP working solutions at 5X final concentration were prepared in 50 mM Bis-Tris pH 6.0, 50 mM NaCl, and 0.01% Tween 20. PTP working solution (20 μL) was dispensed into the microplate and incubated with compound for 20 min at RT. In addition, 5X DiFMUP working solutions were prepared for final concentrations corresponding to the respective *K*_m_ value for STEP (3 μM) or PTP1B (25 μM), respectively. The reaction was initiated by addition of 5 μL DiFMUP working solutions. Fluorescence intensity was measured in a kinetic mode as described above. IC_50_ values were calculated from the corrected initial rates by nonlinear regression using the program GraphPad Prism (version 8, GraphPad Software, Inc., San Diego, CA, USA). 

## Figures and Tables

**Table 1:** Chemical structures of powder-confirmed hits with sub-50 μM activity against STEP_46_. IC_50_ values and corresponding dose-response curves with STEP_46_ and PTP1B are shown.

Substance ID	Structure	STEP_46_ IC_50_, µM	STEP IC_50_ Curve, µM	PTP1B IC_50_, µM	PTP1B IC_50_ Curve, µM
**SBI-6066**	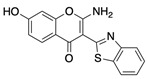	**5.0**	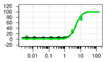	**>100**	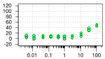
**SBI-2136**	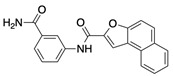	**5.3**	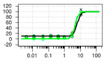	**14**	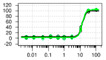
**SBI-6083**	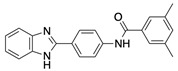	**5.4**	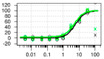	**>100**	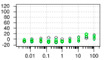
**SBI-0743**	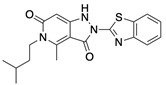	**5.5**	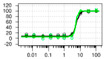	**22**	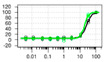
**SBI-0738**	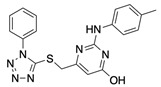	**5.7**	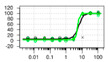	**6.1**	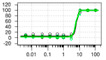
**SBI-1359**	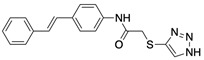	**7.0**	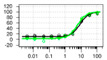	**28**	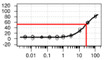
**SBI-8771**	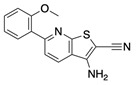	**16**	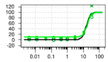	**79**	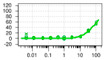
**SBI-6727**	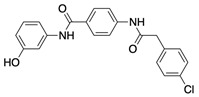	**18**	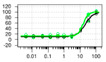	**57**	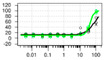
**SBI-7297**	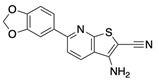	**32**	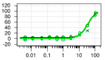	**>100**	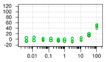
**SBI-8929**	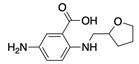	**33**	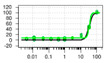	**20**	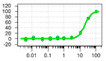

## Abbreviations

Aβ	amyloid-β peptide
AD	Alzheimer’s disease
ADME	absorption, distribution, metabolism, and excretion
AMPA	α-amino-3-hydroxy-5-methyl-4-isoxazolepropionic a
BSA	bovine serum albumin
CNS	central nervous system
DiFMUP	6,8-difluoro-4-methylumbelliferyl phosphate
DMSO	dimethyl sulfoxide
DTT	dithiothreitol
ECFP	extended-connectivity FingerPrint
ERK	extracellular signal-regulated kinase
HTS	high-throughput screening
KIM	kinase-interaction motif
KO	knockout
LTD	long-term depression
LTP	long-term potentiation
MAPK	mitogen-activated protein kinase
Ni-NTA	nickel-nitrilotriacetic acid
NMDA	*N*-methyl-*D*-aspartate
PP1	protein phosphatase 1
PROTAC	proteolysis targeting chimeras
PSD	postsynaptic density
PTK	protein tyrosine kinase
PTP	protein tyrosine phosphatase
PTS	protein thermal shift
RT	room temperature
SAR	structure activity relationship
SD	standard deviation
SEM	standard error of the mean
STEP	striatal-enriched tyrosine phosphatase
TCEP	tris(2-carboxyethyl)phosphine
TKI	tyrosine kinase inhibitor
T_m_	melting temperature
